# 1-(3,5-Dichloro­phen­yl)-1*H*-1,2,3,4-tetra­zole

**DOI:** 10.1107/S1600536812001225

**Published:** 2012-01-18

**Authors:** Rajesh G. Kalkhambkar, D. Gayathri, Vivek K. Gupta, Rajni Kant, Yeon Tae Jeong

**Affiliations:** a Department of Chemistry, Karnatak University’s Karnatak Science College, Dharwad 580 001, Karnataka, India; b Department of Physics, Dr M.G.R. Educational and Research Institute, Dr M.G.R. University, Maduravoyal, Chennai 600 095, India; c X-ray Crystallography Laboratory, Post Graduate Department of Physics & Electronics, University of Jammu, Jammu Tawi 180 006, India; d Department of Image Science and Engineering, Pukyong National University, Busan 608 739, Republic of Korea

## Abstract

In the title compound, C_7_H_4_Cl_2_N_4_, the dihedral angle between the tetra­zole and benzene rings is 17.2 (2)°. In the crystal, C—H⋯N inter­actions link the mol­ecules into a flattened helical chain along the *b* axis.

## Related literature

For related structures, see: Baek *et al.* (2012 ▶); Matsunaga *et al.* (1999 ▶); Lyakhov *et al.* (2000 ▶, 2001 ▶). For the synthesis, see: Su *et al.* (2006 ▶).
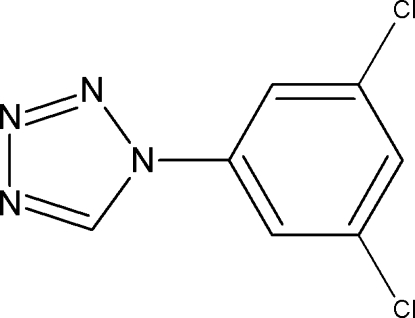


## Experimental

### 

#### Crystal data

C_7_H_4_Cl_2_N_4_
*M* *_r_* = 215.04
Monoclinic, 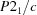

*a* = 3.8362 (2) Å
*b* = 9.0524 (3) Å
*c* = 24.8876 (11) Å
β = 91.956 (4)°
*V* = 863.76 (7) Å^3^
*Z* = 4
Mo *K*α radiation
μ = 0.70 mm^−1^
*T* = 293 K
0.3 × 0.2 × 0.2 mm

#### Data collection

Oxford Diffraction Xcalibur Sapphire3 diffractometer
Absorption correction: multi-scan (*CrysAlis PRO*; Oxford Diffraction, 2010 ▶) *T*_min_ = 0.699, *T*_max_ = 0.869
16772 measured reflections
1692 independent reflections
1451 reflections with *I* > 2σ(*I*)
*R*_int_ = 0.048

#### Refinement

*R*[*F* ^2^ > 2σ(*F* ^2^)] = 0.057
*wR*(*F* ^2^) = 0.114
*S* = 1.17
1692 reflections
118 parameters
H-atom parameters constrained
Δρ_max_ = 0.31 e Å^−3^
Δρ_min_ = −0.25 e Å^−3^

### 

Data collection: *CrysAlis PRO* (Oxford Diffraction, 2010 ▶); cell refinement: *CrysAlis PRO*; data reduction: *CrysAlis PRO*; program(s) used to solve structure: *SHELXS97* (Sheldrick, 2008 ▶); program(s) used to refine structure: *SHELXL97* (Sheldrick, 2008 ▶); molecular graphics: *PLATON* (Spek, 2009 ▶); software used to prepare material for publication: *SHELXL97*.

## Supplementary Material

Crystal structure: contains datablock(s) I, global. DOI: 10.1107/S1600536812001225/is5049sup1.cif

Structure factors: contains datablock(s) I. DOI: 10.1107/S1600536812001225/is5049Isup2.hkl

Supplementary material file. DOI: 10.1107/S1600536812001225/is5049Isup3.cml

Additional supplementary materials:  crystallographic information; 3D view; checkCIF report

## Figures and Tables

**Table 1:** Hydrogen-bond geometry (Å, °)

*D*—H⋯*A*	*D*—H	H⋯*A*	*D*⋯*A*	*D*—H⋯*A*
C1—H1⋯N2^i^	0.93	2.61	3.423 (5)	147
C7—H7⋯N1^i^	0.93	2.53	3.424 (5)	161

Symmetry code: (i) 
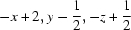
.

**Fig. 1.**
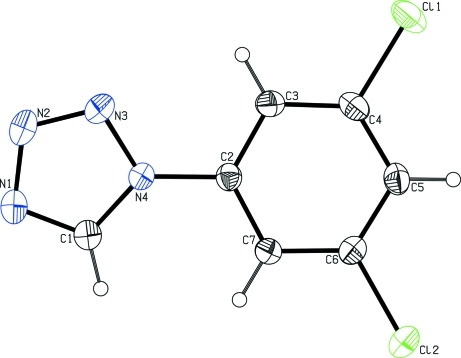
The molecular structure of the title compound, showing 30% probability displacement ellipsoids.

**Fig. 2.**
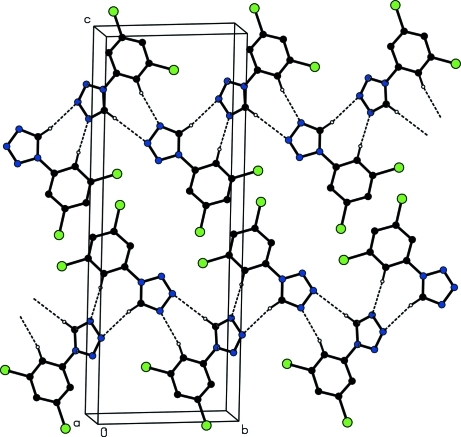
The molecular packing of the title compound, showing intermolecular interactions. For clarity, hydrogen atoms not involved in hydrogen bonding have been omitted.

## supplementary crystallographic information

### Comment

In continuation of our work on tetrazole based heterocycles, we are here in reporting the crystal structure of the title compound.

Bond lengths and angles are comparable with the similar crystal structures (Baek *et al.*, 2012; Lyakhov *et al.*, 2000, 2001; Matsunaga *et al.*, 1999). The tetrazole and phenyl rings are planar, with a maximum out-of-plane deviation of 0.007 (2) Å for each ring (r.m.s. deviation for each ring = 0.005 Å). The two rings are not coplanar with a dihedral angle being 17.2 (2)°. Chlorine atoms Cl1 and Cl2 deviate -0.002 (4) and 0.057 (5) Å, respectively, from the benzene plane. The crystal packing is stabilized by C—H···N intermolecular interactions, wherein atoms C1 and C7 act as a donor to N2 and N1, respectively, generating C(4) and C(6) chains along [010].

### Experimental

The title compound was synthesized from the known procedure reported by Su *et al.* (2006). Fine white diffraction quality crystals were obtained from the slow evaporation of its solution in ethanol.

### Refinement

All H atoms were refined using a riding model, with C—H = 0.93 Å, and with *U*_iso_(H) = 1.2*U*_eq_(C).

### Crystal data

**Table:**

C_7_H_4_Cl_2_N_4_	*F*(000) = 432
*M**_r_* = 215.04	*D*_x_ = 1.654 Mg m^−^^3^
Monoclinic, *P*2_1_/*c*	Mo *K*α radiation, λ = 0.71073 Å
Hall symbol: -P 2ybc	Cell parameters from 7140 reflections
*a* = 3.8362 (2) Å	θ = 4.0–29.0°
*b* = 9.0524 (3) Å	µ = 0.70 mm^−^^1^
*c* = 24.8876 (11) Å	*T* = 293 K
β = 91.956 (4)°	Block, white
*V* = 863.76 (7) Å^3^	0.3 × 0.2 × 0.2 mm
*Z* = 4	

### Data collection

**Table:**

Oxford Diffraction Xcalibur Sapphire3 diffractometer	1692 independent reflections
Radiation source: fine-focus sealed tube	1451 reflections with *I* > 2σ(*I*)
graphite	*R*_int_ = 0.048
Detector resolution: 16.1049 pixels mm^-1^	θ_max_ = 26.0°, θ_min_ = 4.0°
ω scans	*h* = −4→4
Absorption correction: multi-scan (*CrysAlis PRO*; Oxford Diffraction, 2010)	*k* = −11→11
*T*_min_ = 0.699, *T*_max_ = 0.869	*l* = −30→30
16772 measured reflections	

### Refinement

**Table:**

Refinement on *F*^2^	Primary atom site location: structure-invariant direct methods
Least-squares matrix: full	Secondary atom site location: difference Fourier map
*R*[*F*^2^ > 2σ(*F*^2^)] = 0.057	Hydrogen site location: inferred from neighbouring sites
*wR*(*F*^2^) = 0.114	H-atom parameters constrained
*S* = 1.17	*w* = 1/[σ^2^(*F*_o_^2^) + (0.0229*P*)^2^ + 1.3257*P*] where *P* = (*F*_o_^2^ + 2*F*_c_^2^)/3
1692 reflections	(Δ/σ)_max_ < 0.001
118 parameters	Δρ_max_ = 0.31 e Å^−^^3^
0 restraints	Δρ_min_ = −0.24 e Å^−^^3^

### Special details

**Table:**

Geometry. All e.s.d.'s (except the e.s.d. in the dihedral angle between two l.s. planes) are estimated using the full covariance matrix. The cell e.s.d.'s are taken into account individually in the estimation of e.s.d.'s in distances, angles and torsion angles; correlations between e.s.d.'s in cell parameters are only used when they are defined by crystal symmetry. An approximate (isotropic) treatment of cell e.s.d.'s is used for estimating e.s.d.'s involving l.s. planes.
Refinement. Refinement of *F*^2^ against ALL reflections. The weighted *R*-factor *wR* and goodness of fit *S* are based on *F*^2^, conventional *R*-factors *R* are based on *F*, with *F* set to zero for negative *F*^2^. The threshold expression of *F*^2^ > σ(*F*^2^) is used only for calculating *R*-factors(gt) *etc*. and is not relevant to the choice of reflections for refinement. *R*-factors based on *F*^2^ are statistically about twice as large as those based on *F*, and *R*- factors based on ALL data will be even larger.

### Fractional atomic coordinates and isotropic or equivalent isotropic displacement parameters (Å^2^)

**Table:**

	*x*	*y*	*z*	*U*_iso_*/*U*_eq_	
C1	0.8151 (11)	0.8952 (4)	0.22313 (15)	0.0569 (10)	
H1	0.7634	0.8155	0.2450	0.068*	
C2	0.5993 (8)	0.7942 (3)	0.13392 (12)	0.0332 (7)	
C3	0.4810 (8)	0.8412 (4)	0.08358 (13)	0.0389 (7)	
H3	0.4966	0.9397	0.0734	0.047*	
C4	0.3391 (8)	0.7360 (4)	0.04919 (12)	0.0410 (8)	
C5	0.3089 (8)	0.5895 (4)	0.06386 (13)	0.0405 (8)	
H5	0.2125	0.5202	0.0401	0.049*	
C6	0.4258 (8)	0.5491 (3)	0.11477 (13)	0.0378 (7)	
C7	0.5739 (8)	0.6494 (3)	0.15071 (12)	0.0348 (7)	
H7	0.6533	0.6204	0.1848	0.042*	
N1	0.9600 (10)	1.0175 (4)	0.23973 (13)	0.0619 (9)	
N2	0.9905 (10)	1.1003 (4)	0.19516 (15)	0.0682 (10)	
N3	0.8690 (10)	1.0311 (3)	0.15319 (13)	0.0655 (10)	
N4	0.7517 (7)	0.9004 (3)	0.17044 (11)	0.0379 (6)	
Cl1	0.1904 (3)	0.78829 (13)	−0.01453 (4)	0.0645 (3)	
Cl2	0.3786 (3)	0.36798 (10)	0.13489 (4)	0.0639 (3)	

### Atomic displacement parameters (Å^2^)

**Table:**

	*U*^11^	*U*^22^	*U*^33^	*U*^12^	*U*^13^	*U*^23^
C1	0.084 (3)	0.043 (2)	0.042 (2)	−0.014 (2)	−0.0130 (19)	0.0013 (16)
C2	0.0334 (16)	0.0344 (16)	0.0319 (16)	−0.0010 (13)	0.0023 (12)	−0.0025 (13)
C3	0.0421 (18)	0.0371 (18)	0.0375 (17)	−0.0027 (14)	−0.0005 (14)	0.0056 (14)
C4	0.0377 (17)	0.054 (2)	0.0309 (16)	−0.0017 (15)	−0.0021 (13)	0.0045 (15)
C5	0.0388 (18)	0.0460 (19)	0.0367 (17)	−0.0075 (15)	−0.0016 (14)	−0.0061 (15)
C6	0.0403 (17)	0.0319 (16)	0.0414 (18)	−0.0016 (14)	0.0022 (14)	−0.0008 (14)
C7	0.0375 (17)	0.0351 (17)	0.0317 (16)	−0.0022 (13)	−0.0007 (13)	0.0018 (13)
N1	0.087 (3)	0.0454 (18)	0.052 (2)	−0.0120 (18)	−0.0179 (17)	−0.0070 (15)
N2	0.096 (3)	0.0420 (18)	0.065 (2)	−0.0217 (18)	−0.015 (2)	−0.0050 (17)
N3	0.103 (3)	0.0397 (17)	0.053 (2)	−0.0251 (18)	−0.0099 (19)	0.0065 (15)
N4	0.0451 (15)	0.0307 (14)	0.0375 (14)	−0.0040 (12)	−0.0038 (12)	−0.0002 (11)
Cl1	0.0754 (7)	0.0796 (7)	0.0374 (5)	−0.0070 (6)	−0.0160 (4)	0.0084 (5)
Cl2	0.0968 (8)	0.0353 (5)	0.0589 (6)	−0.0147 (5)	−0.0059 (5)	0.0006 (4)

### Geometric parameters (Å, °)

**Table:**

C1—N1	1.300 (5)	C4—Cl1	1.733 (3)
C1—N4	1.326 (4)	C5—C6	1.379 (4)
C1—H1	0.9300	C5—H5	0.9300
C2—C7	1.381 (4)	C6—C7	1.383 (4)
C2—C3	1.384 (4)	C6—Cl2	1.726 (3)
C2—N4	1.434 (4)	C7—H7	0.9300
C3—C4	1.380 (4)	N1—N2	1.347 (5)
C3—H3	0.9300	N2—N3	1.291 (4)
C4—C5	1.382 (5)	N3—N4	1.342 (4)
			
N1—C1—N4	110.3 (3)	C4—C5—H5	121.0
N1—C1—H1	124.9	C5—C6—C7	122.3 (3)
N4—C1—H1	124.9	C5—C6—Cl2	119.0 (2)
C7—C2—C3	122.7 (3)	C7—C6—Cl2	118.7 (2)
C7—C2—N4	118.4 (3)	C2—C7—C6	117.3 (3)
C3—C2—N4	118.8 (3)	C2—C7—H7	121.3
C4—C3—C2	117.4 (3)	C6—C7—H7	121.3
C4—C3—H3	121.3	C1—N1—N2	105.1 (3)
C2—C3—H3	121.3	N3—N2—N1	110.9 (3)
C3—C4—C5	122.2 (3)	N2—N3—N4	106.5 (3)
C3—C4—Cl1	119.3 (3)	C1—N4—N3	107.2 (3)
C5—C4—Cl1	118.4 (3)	C1—N4—C2	131.3 (3)
C6—C5—C4	118.0 (3)	N3—N4—C2	121.5 (3)
C6—C5—H5	121.0		
			
C7—C2—C3—C4	−1.3 (5)	N4—C1—N1—N2	−0.8 (5)
N4—C2—C3—C4	179.3 (3)	C1—N1—N2—N3	0.0 (5)
C2—C3—C4—C5	1.0 (5)	N1—N2—N3—N4	0.7 (5)
C2—C3—C4—Cl1	−179.5 (2)	N1—C1—N4—N3	1.2 (5)
C3—C4—C5—C6	0.0 (5)	N1—C1—N4—C2	179.6 (3)
Cl1—C4—C5—C6	−179.6 (2)	N2—N3—N4—C1	−1.2 (4)
C4—C5—C6—C7	−0.8 (5)	N2—N3—N4—C2	−179.7 (3)
C4—C5—C6—Cl2	178.0 (3)	C7—C2—N4—C1	−16.1 (5)
C3—C2—C7—C6	0.6 (5)	C3—C2—N4—C1	163.4 (4)
N4—C2—C7—C6	180.0 (3)	C7—C2—N4—N3	162.1 (3)
C5—C6—C7—C2	0.5 (5)	C3—C2—N4—N3	−18.4 (5)
Cl2—C6—C7—C2	−178.3 (2)		

### Hydrogen-bond geometry (Å, °)

**Table:**

*D*—H···*A*	*D*—H	H···*A*	*D*···*A*	*D*—H···*A*
C1—H1···N2^i^	0.93	2.61	3.423 (5)	147
C7—H7···N1^i^	0.93	2.53	3.424 (5)	161

Symmetry codes: (i) −*x*+2, *y*−1/2, −*z*+1/2.
